# Long-term actions of interleukin-1β on delay and tonic firing neurons in rat superficial dorsal horn and their relevance to central sensitization

**DOI:** 10.1186/1744-8069-4-63

**Published:** 2008-12-17

**Authors:** Sabrina L Gustafson-Vickers, Van B Lu, Aaron Y Lai, Kathryn G Todd, Klaus Ballanyi, Peter A Smith

**Affiliations:** 1 Centre for Neuroscience and Departments of Pharmacology, Physiology, University of Alberta, Edmonton, Alberta, Canada; 2 Department of Psychiatry, University of Alberta, Edmonton, Alberta, Canada; 3 Faculty of Medicine – Class of 2011, University of British Columbia, Vancouver, BC V6T 1Z3, Canada; 4 Section on Transmitter Signaling, Laboratory of Molecular Physiology, National Institute on Alcohol Abuse and Alcoholism (NIAAA), National Institutes of Health (NIH), 5625 Fishers Lane MSC 9411, Bethesda, MD 20892-9411, USA

## Abstract

**Background:**

Cytokines such as interleukin 1β (IL-1β) have been implicated in the development of central sensitization that is characteristic of neuropathic pain. To examine its long-term effect on nociceptive processing, defined medium organotypic cultures of rat spinal cord were exposed to 100 pM IL-1β for 6–8 d. Interleukin effects in the dorsal horn were examined by whole-cell patch-clamp recording and Ca^2+ ^imaging techniques.

**Results:**

Examination of the cultures with confocal Fluo-4 AM imaging showed that IL-1β increased the change in intracellular Ca^2+ ^produced by exposure to 35–50 mM K^+^. This is consistent with a modest increase in overall dorsal horn excitability. Despite this, IL-1β did not have a direct effect on rheobase or resting membrane potential nor did it selectively destroy any specific neuronal population. All effects were instead confined to changes in synaptic transmission. A variety of pre- and postsynaptic actions of IL-1β were seen in five different electrophysiologically-defined neuronal phenotypes. In putative excitatory 'delay' neurons, cytokine treatment increased the amplitude of spontaneous EPSC's (sEPSC) and decreased the frequency of spontaneous IPSC's (sIPSC). These effects would be expected to increase dorsal horn excitability and to facilitate the transfer of nociceptive information. However, other actions of IL-1β included disinhibition of putative inhibitory 'tonic' neurons and an increase in the amplitude of sIPSC's in 'delay' neurons.

**Conclusion:**

Since spinal microglial activation peaks between 3 and 7 days after the initiation of chronic peripheral nerve injury and these cells release IL-1β at this time, our findings define some of the neurophysiological mechanisms whereby nerve-injury induced release of IL-1β may contribute to the central sensitization associated with chronic neuropathic pain.

## Background

Chronic, intractable neuropathic pain is a major clinical problem. It is initiated by nerve, brain or spinal injury or by complications associated with diseases such as post-herpetic neuralgia, stroke or diabetes. Much of our understanding of the underlying pathophysiology comes from animal models in which experimentally-induced peripheral nerve damage initiates behaviors analogous to the symptoms of human neuropathic pain [1,2]. Manipulations such as sciatic chronic constriction injury (CCI) produce aberrant spontaneous activity in primary afferent fibers [3]. This promotes the release of various 'pain mediators' both from primary afferent terminals and activated spinal microglia [4]. These mediators promote an enduring increase in dorsal horn excitability that underlies the 'central sensitization' which characterizes neuropathic pain.

The cytokine, interleukin 1β (IL-1β) is of special interest in this regard as it is secreted under conditions associated with pain and hyperalgesia [5-7] and is elevated in the cerebral spinal fluid of chronic pain patients [8]. In mice, neutralizing antibodies to interleukin 1-receptor reduce pain behavior associated with experimental neuropathy [9]. Moreover, deletion of IL-1 receptor type 1 or transgenic overexpression of the naturally occurring IL-1 receptor antagonist (IL-1RA) delay the onset and severity of pain associated with peripheral nerve injury [10]. Similar effects are seen in animals lacking the IL-1β gene [11,12].

The matrix metalloproteases, MMP9 and MMP2 have recently been implicated in the onset and maintenance of neuropathic pain [13] and IL-1β identified as a vital downstream effector of their action. It has also been reported that the frequency and amplitude of spontaneous EPSC's (sEPSC) is increased and the frequency and amplitude of spontaneous IPSC's (sIPSCs) is decreased following a 4 min application of 600 pM IL-1β to neurons in the superficial dorsal horn [14]. Although these findings provide information on the acute spinal actions of a relatively high concentration of IL-1β [15], this methodology may not be appropriate for understanding the role of interleukins in central sensitization. There are three reasons for this:

First, in Sprague Dawley rats, spinal microglial activation in response to peripheral nerve injury peaks between days 3 and 7 and returns to normal after about 28 days [4,16] and IL-1β levels continue to increase for at least 35 days [17]. It is likely that microglial-derived IL-1β is involved in the induction phase of central sensitization whereas the maintenance of sensitization may involve astrocyte – derived interleukin [18]. This means that spinal neurons *in vivo *are exposed to elevated interleukin levels for several weeks following nerve injury. It is therefore questionable whether their response to an acute, transient application of IL-1β [14] is relevant to the process of pain centralization which takes days or weeks to develop. This is of particular concern because the neuronal actions of IL-1β are time-dependent. For example, acute (5 min) application of IL-1β reduces Na^+ ^channel currents in primary afferent neurons whereas longer-term (24 h) application has the opposite effect; it increases the current [19]. This latter effect likely involves altered gene expression [20].

Second, random sampling of superficial dorsal horn neurons with patch electrodes reveals that >30% of neurons exhibit a 'tonic' discharge pattern [21], that has been associated with inhibitory interneurons [22]. It is likely therefore that some of the reported acute excitatory actions of IL-1β [14] were exerted on inhibitory interneurons. This would produce an overall dampening of dorsal horn excitability that would be inconsistent with the proposed role of IL-1β in central sensitization.

Third, freshly isolated spinal cord slices, as used in a previous study of cytokine action [14], are likely to be undergoing an acute inflammatory response associated with microglia activation. Substances released from activated microglia might affect neuronal responses to exogenously-applied cytokine.

To address these issues and to provide additional information on neurophysiological mechanisms by which IL-1β produces a slowly developing increase in spinal cord excitability, we examined the effect of long-term (6–8 d) exposure of spinal neurons to IL-1β. Experiments were done using a defined medium organotypic culture system developed in our laboratory [23,24].

We found that IL-1β did not selectively destroy any specific neuronal population, nor did it have a direct effect on postsynaptic membrane excitability. Its actions were instead confined to changes in synaptic transmission. These changes were not homogeneous throughout the whole neuronal population as different effects were seen on putative inhibitory and excitatory neurons. Because overall dorsal horn excitability appeared to increase, pronociceptive/excitatory effects likely dominated over inhibitory/antinociceptive effects. These findings define some of the long-term neurophysiological mechanisms whereby nerve-injury induced release of IL-1β may contribute to the central sensitization associated with chronic neuropathic pain.

## Methods

All experimental procedures were approved by the University of Alberta Health Sciences Laboratory Animal Policy and Welfare Committee.

### Organotypic Cultures

**Figure 1:**
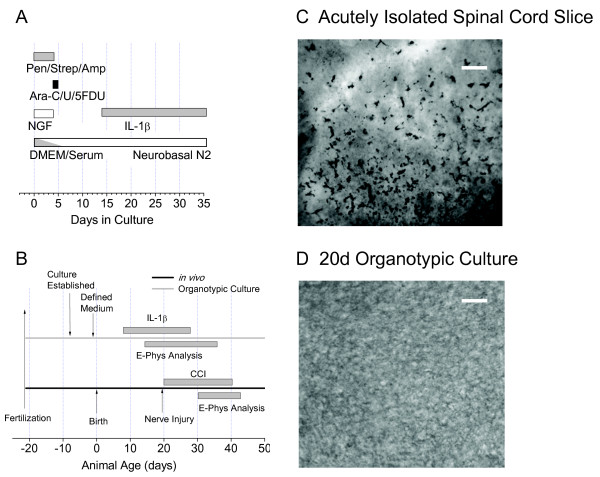
**A. Time course of media exchanges in spinal cord organotypic cultures, establishment of defined medium and time course of IL-1β application**. Nerve Growth Factor (NGF) was 20 ng/ml. Serum was progressively removed by successive dilutions during exchanges of medium. Pen/Strep/Amp = 5 units/mL penicillin G, 5 units/mL streptomycin, and 12.5 ng/mL amphotericin B, Ara-C/U/5 FDU = uridine, cytosine-β-D-arabino-furanoside (AraC), and 5-fluorodeoxyuridine (all at 10 μM). **B**. Comparison of time course of present IL-1β studies with previous studies of sciatic chronic constriction injury (CCI) [21]. Time scale in days refers to age of experimental animals. **C**. Photomicrograph obtained from an acutely isolated spinal cord slice. The central canal appears as the light area just to the right of the upper centre of the picture. The ventral surface of the cord is to the left **D**. Photomicrograph obtained from an organotypic culture, Sections in **C **and **D **both immuno-reacted for the microglial marker Iba-1. Calibration bar in both plates is 50 μm.

Defined medium organotypic cultures of prenatal Sprague-Dawley rat spinal cord were prepared as described previously using a roller drum technique [23,24] The schedule of medium changes is depicted schematically in Fig 1A. Cultures were maintained until the neurons therein attained an age comparable to those in the juvenile rats in which we studied the effects of sciatic chronic constriction injury (CCI) [21,24] (Fig 1B see discussion). Slices were superfused at ~22°C with 95%O_2_-5% CO_2 _saturated artificial cerebrospinal fluid which contained (in mM): 127 NaCl, 2.5 KCl, 1.2 NaH_4G_O_4_, 26 NaHCO_3_, 1.3 MgSO_4_, 2.5 CaCl_2_, 25 D-glucose, pH 7.4 (320–325 mOsm). IL-1β (Calbiochem, Hornby, ON, Canada) treatments were initiated 14 to 28 d after the start of culture. Serum-free medium containing IL1-β was exchanged 4 d thereafter and recordings made on days 6–8. IL-1β stocks, dissolved in bovine serum albumin (BSA) were diluted in medium containing 0.1% BSA. The control group contained the same amount of BSA as the IL-1β group.

### Acute spinal cord slices, Iba-1 staining and IL-1b Measurements

Acute spinal cord slices were prepared from 20–40 d male Sprague-Dawley rats as previously described [21]. These, as well as organotypically cultured slices, were fixed in 10% formalin solution for 5 minutes Fixed slices were then incubated for 30 mins in a blocking solution containing 10% horse serum, 0.03% hydrogen peroxide and 2.5% Triton in PBS. After blocking, the slices were incubated in a primary antibody solution containing 1:1000 anti-Iba1 (Wako, #01-1974) which labels microglia, 1% horse serum, and 2.5% Triton in PBS at 4°C overnight. The slices were then washed and incubated with the secondary antibody, 1:200 biotinylated IgG 1:200 (Serotec), for 30 minutes followed by further washings and incubation with HRP-conjugated-strepavidin (1:200, Vector Labs., Burlingame, CA, USA) for 30 minutes (both were diluted in PBS containing 1% horse serum). The labeling was visualized using diaminobenzidine-hydrogen peroxide (Sigma). IL-1β ELISA kits (R&D systems) were obtained from Cedarlane Laboratories, Burlington, ON, Canada and ELISA procedures carried out according to the manufacturer's protocol.

### Electrophysiology

Whole-cell recordings were made under infrared differential interference contrast optics. Neurons chosen for recording were located 0.5 – 2 mm from the dorsal surface of the slices [23,24]. Data were acquired from neurons that exhibited action potentials > 65 mV in amplitude and were analyzed using pCLAMP 9.0 (Molecular Devices, Burlingame, CA, USA). The solution in patch pipettes contained (in mM): 130 D-gluconic acid, 2 CaCl_2_, 10 HEPES, 10 EGTA, 4 Mg-ATP, 0.3 Na-GTP, 0.2% Biocytin, adjusted to pH 7.2 with KOH (290–300 mOsm). Spontaneous EPSC's (sEPSC) were recorded at -70 mV and spontaneous IPSC's (sIPSC) at 0 mV. Mini Analysis Program (Synaptosoft, Decatur, GA, USA) was used to prepare cumulative probability plots. Up to 100 spontaneous synaptic events from each neuron were used for analysis (up to 200 sIPSC's were analyzed in delay neurons). Cumulative probability plots and bar graphs were constructed for data from 4 to 8 neurons. This meant that similar total numbers of events were sampled from each neuron type in the control and IL-1β treated groups. As in our previous work, both t-tests of means ± SE and the Kolmogorov-Smirnov two-sample test (K-S test) were used to compare distributions of amplitudes and inter-event intervals of synaptic events in the control and experimental situations [21,23,24]. Distributions were considered statistically significant if p < 0.05.

### Confocal Ca^2+ ^Imaging

Cultures were challenged with 20, 35 or 50 mM K^+ ^and the resulting elevation of intracellular Ca^2+ ^concentration used as an index of overall increase in excitability. Ca^2+ ^imaging was carried out as described previously [24,25] using Fluo-4 AM (TEF Labs Inc. Austin, Texas, USA). Fluorescence signals were visualized with a confocal microscope equipped with an argon (488 nm) laser and filters (20× XLUMPlanF1-NA-0.95 objective; Olympus FV300, Carsen group, Markham, Ontario, Canada). Image files were stored on disk for off-line analysis. Selected regions of interest were drawn around distinct cell bodies and traces of time course of change of fluorescence intensity were generated with FluoView v.4.3 (Olympus). Neurons out of the plane of focus were rejected for analysis and data only collected from those that responded reversibly to a high K^+ ^challenge. Data were collected from 4–10 neurons per culture.

## Results

### Microglial status in acute and organoypically-cultured spinal cord slices

Removal and transverse sectioning of the spinal cord to prepare acute spinal cord slices would be expected to produce profound microglial activation. To verify this, we used a marker for the microglial-specific calcium binding protein, Iba1 [26]. Although this protein is present in resting microglia, it is upregulated when they are activated [27]. Fig 1C shows numerous rod-shaped, amoeboid and round cells positive for Iba-1 indicating microglia/macrophages at various stages of activation in an acutely isolated spinal cord slice. This slice was fixed approximately 80 min after removal from the animal and subject to the normal isolation and stabilization processes used in our whole-cell recording protocol [21]. By contrast, the organotypic section displayed in Fig 1D shows very few immuno-positive cells suggesting little microglial/macrophage activation.

### IL-1β Alters Overall Network Excitability

Measured concentrations of IL-1β in dorsal root ganglia or spinal cord of rats exhibiting injury-related pain range from 60 pM [28] to 3 nM [13]. A standard ELISA for IL-1β revealed that the resting concentration of IL-1β in our control cultures was between 4 and 20 pM. We therefore elected to apply cytokine at 100 pM, this yielded a final concentration of 120 pM in the culture medium (as measured by ELISA).

**Figure 2:**
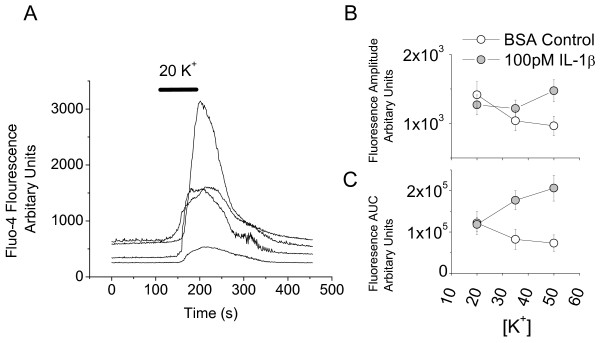
**Effects of 6–8 d exposure of the dorsal hornregion of organotypic cultures to 100 pM IL-1β **A**. Typical time courses of changes in Fluo 4-AM fluorescence, indicating changes in free intracellular [Ca^2+^] in response to raising [K^+^] in superfusate from 2.5 to 20 mM**. Traces are recordings from somata of cells marked as regions of interest for the analysis software. **B **and **C**. Quantification of Fluo-4 fluorescence changes in response to 20, 35 and 50 mM K^+ ^from 26 control and 26 IL-1β treated neurons. Changes in amplitude in response to 50 mM K^+ ^are increased in cytokine-treated slices (p = 0.02). For area under curve (AUC) significant increases are seen for both 35 mM (p < 0.01) and 50 mM K^+ ^(p < 0.001).

Peripheral nerve injury increases the rate of spontaneous and 'pinch-evoked' action potential discharge in the dorsal horn *in vivo *[29]. This presumably reflects altered synaptic transmission [21] and perhaps changes in intrinsic neuronal properties. If IL-1β is involved in central sensitization, it should increase the tendency for the cultures to generate action potentials. Since action potential activity promotes an increase in intraneuronal Ca^2+^, the Ca^2+ ^response to a depolarizing stimulus can be used as an index of the overall excitability of a neuronal network. Thus, to test whether 6–8 d treatment with 100 pM IL-1β increased overall excitability of the dorsal horn, cultures were challenged with 20, 35 and 50 mM K^+ ^and the resulting increases in intracellular [Ca^2+^] monitored using confocal Fluo-4-AM Ca^2+ ^imaging. Twenty-six cells from slices in each condition were analyzed, and both the amplitude and area under the curve (AUC) of the K^+^-induced fluorescence intensity increase were measured. Figure 2A shows fluorescence signals selected from various regions of interest (four neurons selected as per methods section) in response to a 20 mM K^+ ^challenge. With respect to signal amplitude, it was found that the response to 50 mM K^+ ^in IL-1β-treated neurons was significantly greater than that of controls (p = 0.02, Fig 2B). Similarly, the area under the curve of the responses to 35 mM and 50 mM K^+ ^(Fig 2C) was significantly greater in IL-1β-treated cells than in controls (p < 0.01 and <0.001, respectively). This suggests that 6–8 d treatment with 100 pM IL-1β produces a modest increase in overall excitability.

### IL-1β does not affect postsynaptic membrane properties

**Figure 3:**
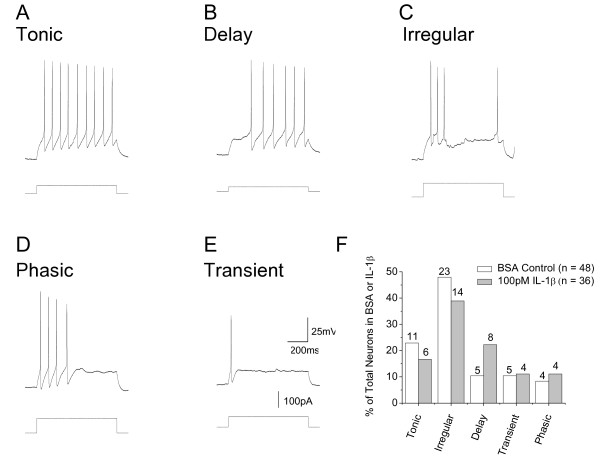
**A – E. Illustrations of discharge pattern of tonic, delay, irregular, phasic and transient neurons in organotypic culture in response to current commands as illustrated**. Membrane potential was set to -60 mV. **F**. Comparison of the percentages of these 5 neuronal types in control (BSA) and cytokine-treated cultures Data from 48 control neurons and 35 interleukin-treated neurons. Numbers over each column refer to number of cells in each category.

We have shown previously that the region of organotypic cultures that was selected for recording [23,24] contains the same five neuronal types that are found in the *substantia gelatinosa *of acutely isolated slices of rat spinal cord [21]. On the basis of their discharge pattern in response to depolarizing current, these are described as tonic, delay, irregular, phasic and transient (Fig 3A–3E). The relative proportion of each cell type stabilizes after about 20 d in culture [23] and this was unaffected by IL-β treatment (Fig 3F, for all groups χ^2 ^> 0.2). The resting membrane potential and rheobase of all five cell types were also unchanged (p values ranged from 0.12 to 0.99, Table 1).

**Table 1:** Effects of IL-1β on Resting Membrane Potential (RMP) and Rheobase in five electrophysiologically defined neuron types in organotypic cultures of rat spinal cord.

	**Tonic**	**Delay**	**Irregular**	**Transient**	**Phasic**
RMP (mV)	-52.5 ± 2.6	-48.6 ± 5.9	-51.3 ± 1.8	-45.0 ± 5.2	-56.3 ± 4.1
**BSA Controls**	*n *= 11	*n *= 5	*n *= 23	*n *= 5	*n *= 4

RMP (mV)	-53.2 ± 4.6	-54.6 ± 3.7	-53.6 ± 1.9	-45.0 ± 5.4	-44.0 ± 4.6
**100 pM IL-1β**	*n *= 6	*n *= 8	*n *= 14	*n *= 4	*n *= 3

Rheobase (pA)	25.0 ± 2.0	61.0 ± 20.0	82.5 ± 12.1	135.0 ± 32.9	118.8 ± 53.2
**BSA Controls**	*n *= 9	*n *= 5	*n *= 18	*n *= 4	*n *= 4

Rheobase (pA)	31.7 ± 10.1	72.1 ± 8.4	70.8 ± 11.9	173.8 ± 65.9	105.0 ± 20.0
**100 pM IL-1β**	*n *= 6	*n *= 7	*n *= 12	*n *= 4	*n *= 2

Data expressed as Means ± SE. p values ranged from 0.12 to 0.99

### Effects of IL-1β on synaptic transmission

IL-1β did not produce a generalized increase or decrease in synaptic transmission but rather caused a series of neuron-type selective changes. Effects on the amplitude and interevent interval of sEPSCs and sIPSCs were analyzed for tonic and delay cells. This is because inhibitory interneurons often exhibit a tonic discharge pattern [22,30] whereas delay neurons may be excitatory [31].

**Figure 4:**
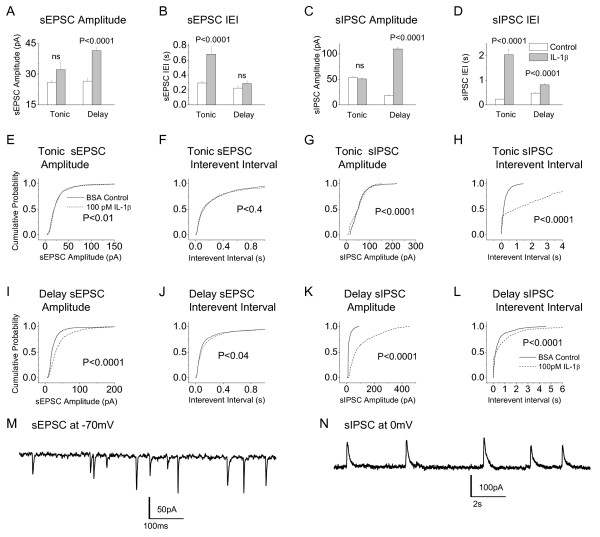
**A – D. Bar graphs illustrating effect of IL-1β on amplitude and interevent interval of sIPSC's and sEPSC's in tonic and delay cells**. Error bars = S.E.M., graphs produced from same data as used for cumulative probability plots. **E – L**. Reanalysis of the data in **A-D **shown as cumulative probability plots. For EPSC's in tonic cells (**E **and **F**), 800 events from 8 neurons analyzed in control (minimum # of events analyzed per cell = 100), 563 events from 6 neurons analyzed in IL-1β (minimum # of events analysed per cell = 63); For IPSC's in tonic cells (**G **and **H**), 600 events from 6 neurons analyzed in control (minimum # of events analyzed per cell = 100), 239 events analyzed in 5 neurons in IL-1β (minimum # of events analysed per cell = 37); For EPSC's in delay cells (**I **and **J**), 699 events analyzed in 7 control neurons (minimum # of events analysed per cell = 99), 500 events analyzed in five neurons in IL-1β (minimum # of events analyzed per cell = 100); For IPSC's in delay cells (**K **and **L**), 751 events analyzed in 4 control neurons (minimum # of events analysed per cell = 151), 1085 events analyzed in 6 neurons in IL-1β (minimum # of events analysed per cell = 85). **M **and **N**. Sample recordings of sEPSCs at -70 mV and sIPSC's at 0 mV.

Effects of IL-1β on sEPSC's and sIPSC's were analyzed in two ways. First, mean values of amplitude and interevent intervals were compared by means of a t-test. Data for tonic and delay cells are shown in Fig 4A–4D. Second, the same data sets were re-plotted as cumulative probabilities (Fig 4E–4L) and compared using the K-S test. The following effects of IL-1β were significant according to *both *analyses:-

1. An increase in the amplitude of sEPSC's (Fig 4A and 4I) and an increase in the interevent interval (decreased frequency) of sIPSC's in delay neurons (Fig 4D and 4L). These actions would be expected to augment synaptic drive to putative excitatory neurons. This would tend to increase overall excitability and to facilitate the transfer of nociceptive information.

2. Large increases in the amplitude of sIPSC's in delay neurons (Fig 4C and 4K) and increases in the interevent interval (decreased frequency) of sIPSC's in tonic neurons (Fig 4D and 4H). Increased inhibition of excitatory cells and disinhibition of inhibitory cells might be expected to reduce overall network excitability.

Sample recordings of sEPSC's at -70 mV and sIPSC's at 0 mV are shown in Figs 4M and 4N.

## Discussion

Spinal microglial activation in response to peripheral nerve injury peaks between days 3 and 7 and returns to normal after about 28 days [4,16] whereas IL-1β levels continue to increase for at least 35 days [17]. Since it is impractical to apply interleukin for this length of time, we opted to apply IL-1β for 6–8 d as as this would still allow us to observe changes resulting from altered gene expression. Data from Ca^2+ ^imaging (Fig 2A–2C) show that 6–8 d exposure to 100 pM IL-1β produces a modest increase in spinal cord excitability. This does not appear to reflect excitotoxic death of inhibitory neurons [32] as comparable numbers of tonic neurons were seen in control and in IL-1β treated slices (Fig 3F). Although there is evidence that 24 h exposure of primary afferent neurons to IL-1β increases excitability by an action on Na^+ ^channels [19], such changes are unlikely to occur following 6–8 d exposure of neurons in the superficial dorsal horn as rheobase was unaffected (Table 1). Since IL-1β promoted clear neuron-type specific alterations in synaptic transmission, it is likely that the modest increase in overall excitability reflected an alteration in the balance of actions of excitatory and inhibitory neurotransmitters.

### Effects of IL-1β on inhibitory synaptic transmission

Acute application of IL-1β potentiates the action of GABA in amygdala [33] and hippocampus [34] yet reduces sIPSC amplitude and GABA sensitivity in the superficial dorsal horn [14]. Acute interleukin application also acts presynaptically in amygdala to reduce neurotransmitter release [33]. This may perhaps reflect suppression of Ca^2+ ^channel conductance [35] in presynaptic terminals. It is difficult to relate these results to ours, as to the best of knowledge, only one other study of the long-term neurophysiological actions IL-1β on central neurons has appeared [36]. The observed increase in interevent interval of sIPSC's (Fig 4D, 4H and 4L) in both tonic and delay cells suggests that some long-term actions of IL-1β at inhibitory synapses are mediated presynaptically. It remains to be determined whether this reflects retraction of inhibitory synapses, a direct action on the neurotransmitter release process, an action on presynaptic Ca^2+ ^channels, a change in discharge frequency of presynaptic action potentials or an alteration in the ability of the terminals to release neurotransmitter.

The increase in sIPSC amplitude in delay cells (Figs 4C and 4K) seen with long-term application of IL-1β is in direct contrast to its aforementioned acute effect on unidentified dorsal horn neurons where sIPSC amplitude is decreased [14]. It is however similar to the long-term action of IL-1β in organotypically cultured hippocampal neurons [36]. Differences in the spinal actions may reflect both the time course and the concentration of applied interleukin. We used 100 pM IL-1β in our long term experiments whereas others examined the acute actions of 600 pM (10 ng/ml) cytokine [14]. This concentration difference may be important because the neuronal effects of 'physiological' concentrations of IL-1β differ from those seen with 'toxic' or 'pharmacological' concentrations [15].

### Effects of IL-1β on excitatory synaptic transmission

Acute applicationof IL-1β has been reported to increase current through AMPA and NMDA receptor-channels and to increase intracellular calcium levels [14,37], it may therefore activate a postsynaptic process akin to long-term potentiation. The increase in sEPSC amplitude that we saw in delay cells (Fig 4A and 4I) could reflect persistence of this acute effect with prolonged IL-1β application.

By contrast, acute (2 min) application of IL-1β has been reported to increase sEPSC frequency in unidentified neurons in the superficial dorsal horn [14]. This effect presumably abates during long-term interleukin application as we saw no appreciable increase in sEPSC frequency (decrease in interevent interval) in either tonic or delay neurons. In fact, an increase in the mean interevent interval in tonic cells was seen (Fig 4B) but this was not at all significant according to the K-S test (P < 0.4 Fig 4F). There was also a increase in interevent interval in delay cells but this was barely significant according to the K-S test (P < 0.04, Fig 4J) and not significant according to the T-test (Fig 4B). A conservative interpretation of these results is that the effects of long-term IL-1β application on sEPSC frequency (Figs 4B, 4F and 4J) play only a small role in determining overall spinal cord excitability.

### Relative importance of inhibitory and excitatory actions

Although the correlation between tonic firing cells and inhibitory function and delay firing cells and excitation is not absolute, several observations support this generalization. For example, studies using a transgenic mouse strain co-expressing enhanced green fluorescent protein and GAD-67 associated the GABA phenotype with initial burst (phasic), gap (irregular) or tonic and not with delay firing patterns [38]. Also, tonic cells often exhibit an 'islet cell' morphology [31] and many islet cells are inhibitory [30]. By contrast, paired recordings in *substantia gelatinosa *have shown that intracellular stimulation of delay firing cells produces excitatory events in postsynaptic neurons [39]. Thus, the IL-1β-induced increase in sEPSC amplitude and the observed decrease in sIPSC frequency in delay neurons (Fig 4A,4D,4I and 4L) would be expected to increase overall dorsal horn excitability. By contrast, the increase in sIPSC amplitude in delay cells (Fig 4C and 4K) implies increased inhibition of putative excitatory neurons. The decreased frequency of sIPSC's in tonic cells (Fig 4D and 4H) would lead to disinhibition of putative inhibitory neurons. Both would be expected to decrease overall dorsal horn excitability. Data from the Ca^2+ ^imaging experiments (Fig 2A–2C) suggest however that excitatory actions dominate as there is a modest increase in overall network activity. It is also likely that changes in synaptic excitation of irregular, phasic and transient neurons contribute to the overall effect of IL-1β. This possibility is difficult to address until much more is known about *substantia gelatinosa *circuitry and the neurotransmitter phenotype of irregular, phasic and transient cells is determined.

### Use of organotypic cultures to study long-term IL-1β effects

As shown in Fig 1B, our experiments with organotypic culture were designed to mimic *in vitro *the effects of CCI *in vivo *[21]. Because of the longevity of the cultures [23], we were able to approximate the duration of cytokine exposure to the time course of microglial activation reported under these circumstances [4]. There has been some discussion as to whether the 20 d rats we used in our *in vivo *experiments [21] are a relevant model for central sensitization as rats of similar age fail to develop allodynia in response to spared nerve injury [40]. In our experimental situation however, rats subject to CCI displayed clear hyperalgesia 5 d post injury and clear allodynia 14 d post injury [21]. We therefore conclude that our treatment protocol for cultures with IL-1β (Fig 1B) is highly pertinent to understanding the pathophysiology of central sensitization *in vivo*.

Although neurons in our cultures exhibit the same range of electrophysiological and morphological phenotypes as those found in acute slices, spontaneous synaptic activity is increased [23,24]. It may therefore be argued that there is some developmental difference in the cultures compared to neurons of similar age in intact animals. Although this argument is difficult to dismiss, other studies of similar cultures have shown that they retain the basic cytoarchitecture of the spinal segment from which they are derived [41,42]. Moreover, the appearance of a variety of developmental markers mirrors that seen *in vivo *[43]. A further advantage of using the cultures is that there is no detectable microglia activation at the time of IL-1β application (Fig 1D compared to Fig 1C). the presence of activated microglia in acutely isolated slices questions their suitability for studies of cytokine action.

## Conclusion

Our findings define some of the basic neurophysiological consequences of long-term exposure of the spinal dorsal horn to 1L-1β. At 100 pM, the cytokine neither promotes death of any particular neuronal phenotype nor does it directly affect membrane excitability. It promotes selective and differential effects on pre- and postsynaptic processes in inhibitory and excitatory neurons that lead to a modest overall increase in dorsal horn excitability. These findings show how IL-1β may contribute to the onset and maintenance of the central sensitization that underlies neuropathic pain.

## Abbreviations

BSA: Bovine serum albumin; CCI: Chronic Constriction Injury (of sciatic nerve); IL-1β: Interleukin 1β; sEPSC: Spontaneous excitatory postsynaptic current; sIPSC: Spontaneous inhibitory postsynaptic current.

## Competing interests

The authors declare that they have no competing interests.

## Authors' contributions

SLG-V carried out the electrophysiological and imaging aspects of the experimental work and data interpretation and wrote up work as an MSc thesis at the University of Alberta, VBL trained SLG-V in the experimental techniques and data interpretation and participated in early experiments and their analysis, AYL carried out ELISA analysis of interleukin levels and Iba-1 staining of slices in KGT's laboratory. KB trained SLG-V in Flou-4 AM confocal Ca^2+ ^imaging and participated in all experiments of this type, PAS designed the study and acquired CIHR funding. He also prepared the final version of the manuscript and redesigned the figures from their much more extensive form presented in SLG-V's MSc thesis. All authors participated in reviewing, discussing and updating the manuscript, all have read and approved the final version.
